# Supplementary material for: Content-rich biological network constructed by mining PubMed abstracts
Source: BMC Bioinformatics. 2004 Oct 8;5:147. doi: 10.1186/1471-2105-5-147 (PMC528731; doi:10.1186/1471-2105-5-147)
Supplement: Additional File 2 — The original results of the above study (non-essential files are deleted to keep the file size under the limit set by BMC bioinformatics). [file 1471-2105-5-147-S2.bz2 › chilibotAdditionalFile2/dip40/744ID10419942E18123/html/TOLQ_TOLR.html]

 


 **TOLQ** and **TOLR** 
  
Found 36 abstracts in PubMed, retrieved 36.  
 

 What does Google say? 
 PDF only 
| .edu only 

---

**Interactive relationship** (e.g. stimulation, inhibition, etc)

**Neutral relationship**- Role of  **TolR**  N terminal, central, and C terminal domains in dimerization and interaction with TolA and  **tolQ** .  Ref: 10419942 J Bacteriol, 1999
- These results confirmed the essential role of the transmembrane domain of TolA in triggering interactions with  **TolQ**  and  **TolR** .  Ref: 9851983 J Bacteriol, 1998
- Since these two TolA derivatives still formed specific complexes with  **TolR**  derivatives still formed specific complexes with  **TolR**  and  **TolQ** , we concluded that the TolA N terminal domain interacted with these proteins.  Ref: 7744737 J Biol Chem, 1995
- This gene is located between fii and tolA, and we propose the designation of tolQRA for this cluster in which  **tolQ**  is the former fii gene and  **tolR**  is the new open reading frame.  Ref: 3294803 J Bacteriol, 1987
- ExbB  **TolR**  and ExbD  **TolQ**  mixtures showed little if any complementation of exbB exbD and  **tolQ**   **tolR**  mutants indicating coevolution of ExbB with ExbD and  **TolQ**  with  **TolR** .  Ref: 8316079 Mol Microbiol, 1993
- Analysis of sequence alignments of  **TolQ**  and  **TolR**  homologues from numerous Gram negative bacterial genomes, together with analyses of the different  **tolQ**   **tolR**  mutants, revealed that the TM domains of  **TolQ**  and  **TolR**  present structural and functional homologies not only to ExbB and ExbD of the TonB system but also with MotA and MotB of the flagellar motor.  Ref: 11722743 Mol Microbiol, 2001
- These complexes were absent in a  **tolQ**  strain, while only the 65 kDa complex was absent in a  **tolR**  strain.  Ref: 7744737 J Biol Chem, 1995
- The membrane topologies of  **TolQ**  and  **TolR**  are similar to those proposed for ExbB and ExbD, respectively, which is consistent with the partial functional substitution between ExbB and  **TolQ**  and between ExbD and  **TolR** .  Ref: 8331075 J Bacteriol, 1993
- Suppressor mutant analyses have localized the  **TolQ**  TolA interaction to the first transmembrane domain of  **TolQ**  and have shown that the third transmembrane domain of  **TolQ**  interacts with the transmembrane domain of  **TolR** .  Ref: 10419942 J Bacteriol, 1999

**Non-interactive relationship** (e.g. studied together, co-existance, homology, etc.)

- Mutations affecting the function of TolA resulted in a lack or an alteration in TolA  **TolQ**  or  **TolR**  TolA interactions but did not affect the formation of  **TolQ**   **TolR**  complexes.  Ref: 9851983 J Bacteriol, 1998
- Energy minimization of atomic models of the wild type and mutated forms of  **TolQ**  III and  **TolR**  I suggests that the deleterious effect of the A177V substitution arises from a direct steric hindrance of this residue with neighboring transmembrane segments,.  Ref: 7853390 J Mol Biol, 1995
- Activity of ExbB ExbD via TolA was higher in cells lacking TonB, and activity of  **TolQ**   **TolR**  via TonB was increased when TolA was missing.  Ref: 8316079 Mol Microbiol, 1993
- Transcriptional analysis of the tol genes in P. aeruginosa revealed that the  **tolQ**  and  **tolR**  genes are cotranscribed as an approximately 1.5 kb transcript.  Ref: 8955385 J Bacteriol, 1996
- Sequence homology and mutual functional substitution of ExbB ExbD and  **TolQ**   **TolR**  suggest the evolution of the two import systems from a single import system.  Ref: 8316079 Mol Microbiol, 1993
- **TolQ** ,  **TolR** , and TolA are located in the cytoplasmic membrane where they interact with each other via their transmembrane domains.  Ref: 10633120 J Bacteriol, 2000
- This cluster was found to consist of seven genes in the order orf1  **tolQ**   **tolR**  tolA tolB oprL orf2.  Ref: 10735848 J Bacteriol, 2000
- In this study, we show that both  **TolQ**  and  **TolR**  proteins are essential for the TolA Pal interaction.  Ref: 11722743 Mol Microbiol, 2001
- They showed strong homologies to the  **TolQ**  and  **TolR**  proteins, respectively, which are involved in uptake of group A colicins and infection by filamentous bacteriophages.  Ref: 2670903 J Bacteriol, 1989
- In the present study we cloned and mutagenized the orf1,  **tolQ** ,  **tolR** , tolA, and tolB genes from P. putida KT2440, which were located upstream of the oprL gene.  Ref: 10940016 J Bacteriol, 2000
- The  **tolQ** ,  **tolR** , and tolA genes from Pseudomonas aeruginosa PAO were cloned using degenerate oligonucleotide PCR primers designed based on conserved transmembrane regions of Escherichia coli  **TolQ**  and  **TolR**  and E. coli and Pseudomonas putida ExbB and ExbD.  Ref: 8955385 J Bacteriol, 1996
- The  **TolQ**   **TolR**  proteins energize TolA and share homologies with the flagellar motor proteins MotA MotB.  Ref: 11722743 Mol Microbiol, 2001
- Group B colicins, ferric siderophores and vitamin B12 are taken up via the TonB ExbB ExbD, group A colicins via the TolA  **TolQ**   **TolR**  system.  Ref: 8316079 Mol Microbiol, 1993
- Cross linking experiments have shown that the TolA transmembrane domain interacts with  **TolQ**  and  **TolR** .  Ref: 10419942 J Bacteriol, 1999
- The deduced amino acid aa sequence of the H. influenzae  **TolQ** ,  **TolR** , TolA and TolB show 6 1 and 62% identity with Escherichia coli TolQRAB proteins, respectively.  Ref: 8921895 Gene, 1996
- Transmembrane domains of  **TolQ** ,  **TolR**  and TolA interact in the cytoplasmic membrane, while TolB and Pal form a complex near the outer membrane.  Ref: 9701827 Mol Microbiol, 1998
- The  **TolR**  transmembrane domain was shown to be involved in the TolA  **TolR**  and  **TolQ**   **TolR**  interactions, while  **TolR**  central and C terminal domains appeared to be involved in  **TolR**  dimerization.  Ref: 10419942 J Bacteriol, 1999
- **TolQ**  and  **TolR**  span the inner membrane with three and with one alpha helical segments, respectively.  Ref: 7853390 J Mol Biol, 1995
- Six of these new genes encode different homologues of the  **TolR** , TolB, and  **TolQ**  transport proteins, suggesting that adventurous motility is dependent on biopolymer transport.  Ref: 12828649 Mol Microbiol, 2003
- TolA,  **TolQ** , and  **TolR**  are inner membrane proteins, TolB is a periplasmic protein, and Pal, the peptidoglycan associated lipoprotein, is anchored to the outer membrane.  Ref: 11790745 J Bacteriol, 2002
- Evolutionary relationship of uptake systems for biopolymers in Escherichia coli cross complementation between the TonB ExbB ExbD and the TolA  **TolQ**   **TolR**  proteins.  Ref: 8316079 Mol Microbiol, 1993
- This change requires  **TolQ** ,  **TolR** , and a functional TolA N terminal domain.  Ref: 11418549 J Bacteriol, 2001
- Group A colicins need proteins of the Escherichia coli envelope Tol complex TolA, TolB,  **TolQ**  and  **TolR**  to reach their cellular target.  Ref: 9466263 Mol Microbiol, 1998
- Bacteria carrying tolA,  **tolQ**  and  **tolR**  mutations kept their colicin insensitivity as spheroplasts, just as the resistant ones.  Ref: 12094727 Folia Microbiol Praha , 2002
- The interactions of  **TolR**  with TolA and  **TolQ**  were confirmed,  **TolR**  was shown to dimerize, and the resulting dimer was shown to interact with  **TolQ** .  Ref: 10419942 J Bacteriol, 1999
- They first interact with receptors located at the surface of the outer membrane and are then transferred across the cell envelope in a process that requires energy and depends upon accessory proteins TolA, TolB, TolC,  **TolQ** ,  **TolR**  which might play a role similar to that of the secretory apparatus of eukaryotic and prokaryotic cells.  Ref: 3139035 Biochim Biophys Acta, 1988
- Characterization of the tol pal region of Escherichia coli K 12 translational control of  **tolR**  expression by  **TolQ**  and identification of a new open reading frame downstream of pal encoding a periplasmic protein.  Ref: 8763928 J Bacteriol, 1996
- The Tol Pal system of Escherichia coli is composed of the YbgC,  **TolQ** , TolA,  **TolR** , TolB, Pal and YbgF proteins.  Ref: 11994151 Mol Microbiol, 2002
- **TolQ** ,  **TolR**  and TolA are membrane proteins involved in maintaining the structure of Escherichia coli cell envelope.  Ref: 7853390 J Mol Biol, 1995
- These observations strongly suggest that  **TolR**  and  **TolQ**  interact via their transmembrane segments.  Ref: 7853390 J Mol Biol, 1995
- The N terminal transmembrane domain of TolA anchors the protein to the cytoplasmic membrane and interacts with  **TolQ**  and  **TolR** .  Ref: 9851983 J Bacteriol, 1998
- Import of biopolymers into Escherichia coli nucleotide sequences of the exbB and exbD genes are homologous to those of the  **tolQ**  and  **tolR**  genes, respectively.  Ref: 2670903 J Bacteriol, 1989
- The resulting PCR product was used as a probe to isolate a 6.5 kb DNA fragment containing P. aeruginosa  **tolQ** ,  **tolR** , and tolA.  Ref: 8955385 J Bacteriol, 1996
- Interestingly in all the cases examined the  **tolQ**  and  **tolR**  genes or their homologues appear to be present as a pair, with a potential for a tight translational regulation.  Ref: 11200223 J Mol Microbiol Biotechnol, 2001
- The  **TolQ** ,  **TolR** , TolA, TolB, and Pal proteins appear to function in maintaining the integrity of the outer membrane, as well as facilitating the uptake of the group A colicins and the DNA of the infecting filamentous bacteriophages.  Ref: 8763928 J Bacteriol, 1996
- DNA transfer requires the chromosomally encoded TolA,  **TolQ** , and  **TolR**  cytoplasmic membrane proteins.  Ref: 9537368 J Bacteriol, 1998
- This fragment was also detected in Delta  **tolQ** , Delta  **tolR** , and tolA H22P mutants but, in contrast to the parental strain, was also seen in the absence of uncouplers.  Ref: 11418549 J Bacteriol, 2001
- These data demonstrate that TolA interacts directly with  **TolR**  and  **TolQ** .  Ref: 7744737 J Biol Chem, 1995
- The TolA, TolB,  **TolQ** , and  **TolR**  proteins are involved in maintaining the integrity of the Escherichia coli outer membrane and in the import of group A colicins and filamentous phage DNA.  Ref: 7744737 J Biol Chem, 1995
- The nucleotide sequence of a 2.9 kb DNA fragment containing the  **tolQ** ,  **tolR** , and tolA genes was determined.  Ref: 8955385 J Bacteriol, 1996
- TolA N terminal domain interacts with  **TolQ**  and  **TolR**  proteins.  Ref: 7744737 J Biol Chem, 1995
- However, strains with mutations in both tcpA and either  **tolQ** ,  **tolR** , or tolA were completely resistant to CTXphi infection.  Ref: 10692381 J Bacteriol, 2000
- Mutations in  **tolQ** ,  **tolR** , tolA, tolB, or pal genes result in sensitivity to bile salts and the leakage of periplasmic proteins.  Ref: 10633120 J Bacteriol, 2000
- **TolQ**   **TolR**  interacted through TonB, and ExbB ExbD interacted through TolA with the outer membrane receptors and colicins.  Ref: 8316079 Mol Microbiol, 1993
- This infection is dependent on an intact particle and requires the products of the  **tolQ** ,  **tolR** , and tolA genes of the bacteria.  Ref: 3053655 J Bacteriol, 1988
- It is organized into two complexes, one near the outer membrane between TolB and PAL and one in the cytoplasmic membrane between TolA,  **TolQ** , and  **TolR** .  Ref: 10419942 J Bacteriol, 1999
- In this study, we demonstrate that the genes  **tolQ** ,  **tolR** , and tolA, but not tolB, tolC, and ompF, are required for the internalization of cloacin DF13 and they are not involved in the transport of ferric aerobactin.  Ref: 8419302 J Bacteriol, 1993
- TolA,  **TolQ** , and  **TolR**  are localized in the inner membrane while TolB is periplasmic, although a small amount of membrane associated TolB is always found.  Ref: 7744737 J Biol Chem, 1995
- Membrane topologies of the  **TolQ**  and  **TolR**  proteins of Escherichia coli inactivation of  **TolQ**  by a missense mutation in the proposed first transmembrane segment.  Ref: 8331075 J Bacteriol, 1993
- Entry of the hybrid toxin was also shown to depend on the products of tolA,  **tolQ** , and  **tolR**  which are required both for phage f1 infection and for entry of E colicins.  Ref: 3045089 J Bacteriol, 1988
- Analysis of polar mutations, reverse transcriptase PCR assays, and transcriptional fusion constructs with a promoterless lacZ gene revealed that the genes are arranged in two operons orf1  **tolQ**   **tolR**  tolA tolB and oprL orf2.  Ref: 12486055 J Bacteriol, 2003
- Energy dependent conformational change in the TolA protein of Escherichia coli involves its N terminal domain,  **TolQ** , and  **TolR** .  Ref: 11418549 J Bacteriol, 2001
- When the tol strains were transformed with plasmids encoding  **TolR**  or  **TolQ** , the specific complexes were restored.  Ref: 7744737 J Biol Chem, 1995
- The  **TolQ**  and  **TolR**  proteins of Escherichia coli are required for the uptake of group A colicins and for infection by filamentous phages.  Ref: 8331075 J Bacteriol, 1993
- The Tol Pal system of the Escherichia coli envelope is formed from the inner membrane  **TolQ** ,  **TolR**  and TolA proteins, the periplasmic TolB protein and the outer membrane Pal lipoprotein.  Ref: 11115123 Mol Microbiol, 2000
- Moreover, immunoprecipitation experiments with the antiserum directed against TolA indicated that  **TolQ**  and  **TolR**  were co immunoprecipitated with TolA after cross linking.  Ref: 7744737 J Biol Chem, 1995
- It was shown that the TolA,  **TolQ** , and  **TolR**  proteins play a critical role in the translocation of group A colicins and filamentous bacteriophage minor coat proteins g3p.  Ref: 11851406 Biochemistry, 2002
- Polar and nonpolar mutations of the P. putida  **tolQ** ,  **tolR** , tolA, and tolB genes were generated in vitro by using the omega Km r interposon, which carries two transcriptional stop signals, or a promoterless xylE cassette, lacking any transcriptional stop signal, respectively.  Ref: 10940016 J Bacteriol, 2000
- The  **TolQ**   **TolR**  TolA proteins form a complex in the cytoplasmic membrane, while TolB and Pal interact near the outer membrane.  Ref: 10474183 FEMS Microbiol Lett, 1999
- In Pseudomonas putida, the seven genes, orf1,  **tolQ** ,  **tolR** , tolA, tolB, oprL, and orf2, which encode the proteins of this complex, are clustered in a 5.8 kb region of chromosomal DNA.  Ref: 12486055 J Bacteriol, 2003
- Genes homologous to  **tolQ**  and  **tolR**  proved to be very widespread being found throughout the eubacteria, and one example in the archea, this distribution argues for an ancient origin of these genes.  Ref: 11200223 J Mol Microbiol Biotechnol, 2001
- TolA and  **TolR**  are anchored to the inner membrane by a single transmembrane domain,  **TolQ**  is an integral membrane protein with three transmembrane segments, and TolB has recently been found to be periplasmic although it is partially membrane associated.  Ref: 7744736 J Biol Chem, 1995
- Transmembrane domains of  **TolQ** ,  **TolR** , and TolA interact in the cytoplasmic membrane, while TolB and Pal form a complex near the outer membrane.  Ref: 9851983 J Bacteriol, 1998
- Since  **TolQ**  is required for colicin A and E1 uptake, in contrast to  **TolR** , which is necessary only for colicin A, we hypothesized that the tolQ925 mutation might affect an interaction between  **TolQ**  and  **TolR** .  Ref: 7853390 J Mol Biol, 1995
- Isolation and characterization of the Haemophilus influenzae  **tolQ** ,  **tolR** , tolA and tolB genes.  Ref: 8921895 Gene, 1996
- V. cholerae strains with insertion mutations in  **tolQ** ,  **tolR** , or tolA were reduced in their efficiency of CTXphi uptake by 4 orders of magnitude.  Ref: 10692381 J Bacteriol, 2000
- Subsequent translocation of phage DNA requires the chromosomally encoded  **TolQ** ,  **TolR** , and TolA proteins, after the pilus presumably has withdrawn, bringing the phage to the bacterial surface.  Ref: 9335297 J Bacteriol, 1997
- **TolQ** ,  **TolR** , and TolA inner membrane proteins of Escherichia coli are involved in maintaining the stability of the outer membrane.  Ref: 11418549 J Bacteriol, 2001
- Sequence data showed that these genes are clustered in a 6 kb segment of DNA with the gene order orf1  **tolQ**   **tolR**  tolA tolB pal orf2 a newly identified open reading frame encoding a 29 kD9 protein.  Ref: 8763928 J Bacteriol, 1996
- The predicted amino acid sequences of V. cholerae  **TolQ** ,  **TolR** , TolA, and TolB showed significant similarity to the corresponding E. coli sequences.  Ref: 10692381 J Bacteriol, 2000
- Cross complementation between the two systems was demonstrated in that  **tolQ**   **tolR**  mutants transformed with plasmids carrying exbB exbD became sensitive to group A colicins, and exbB exbD mutants transformed with plasmid encoded  **tolQ**   **tolR**  became sensitive to group B colicins.  Ref: 8316079 Mol Microbiol, 1993
